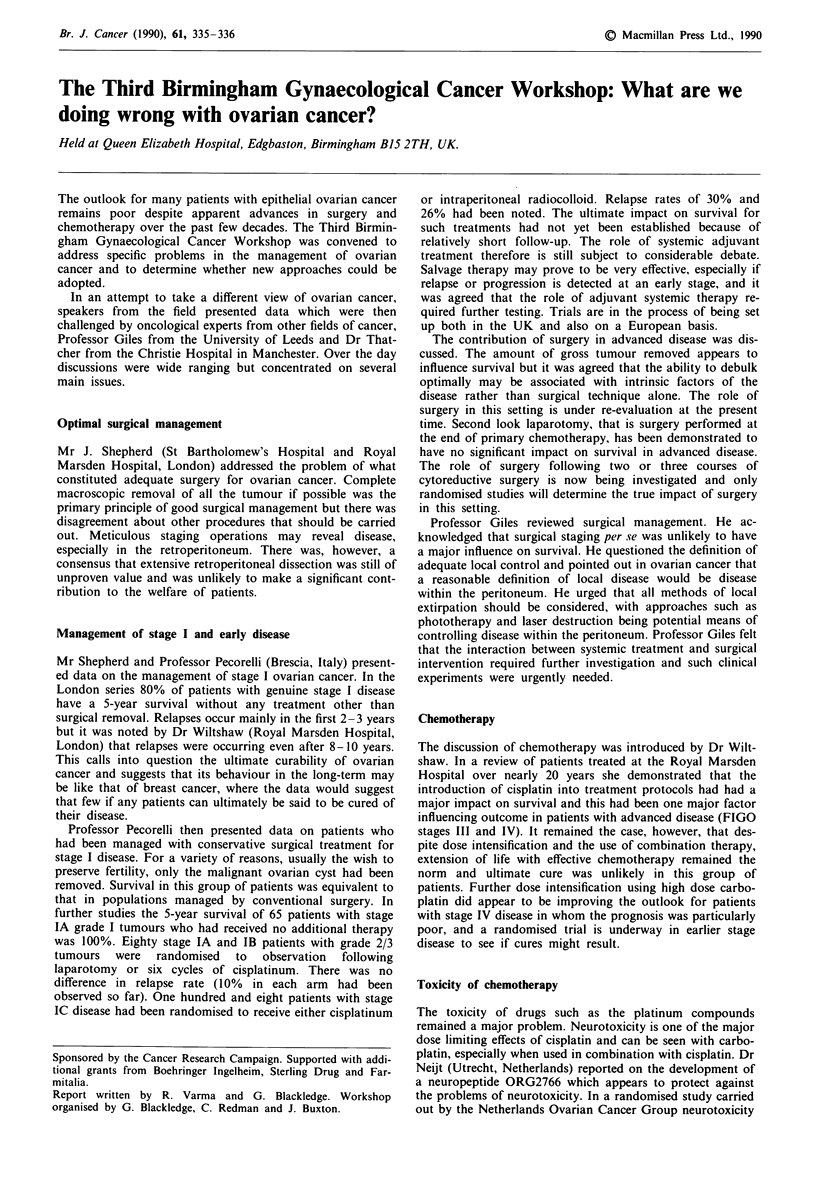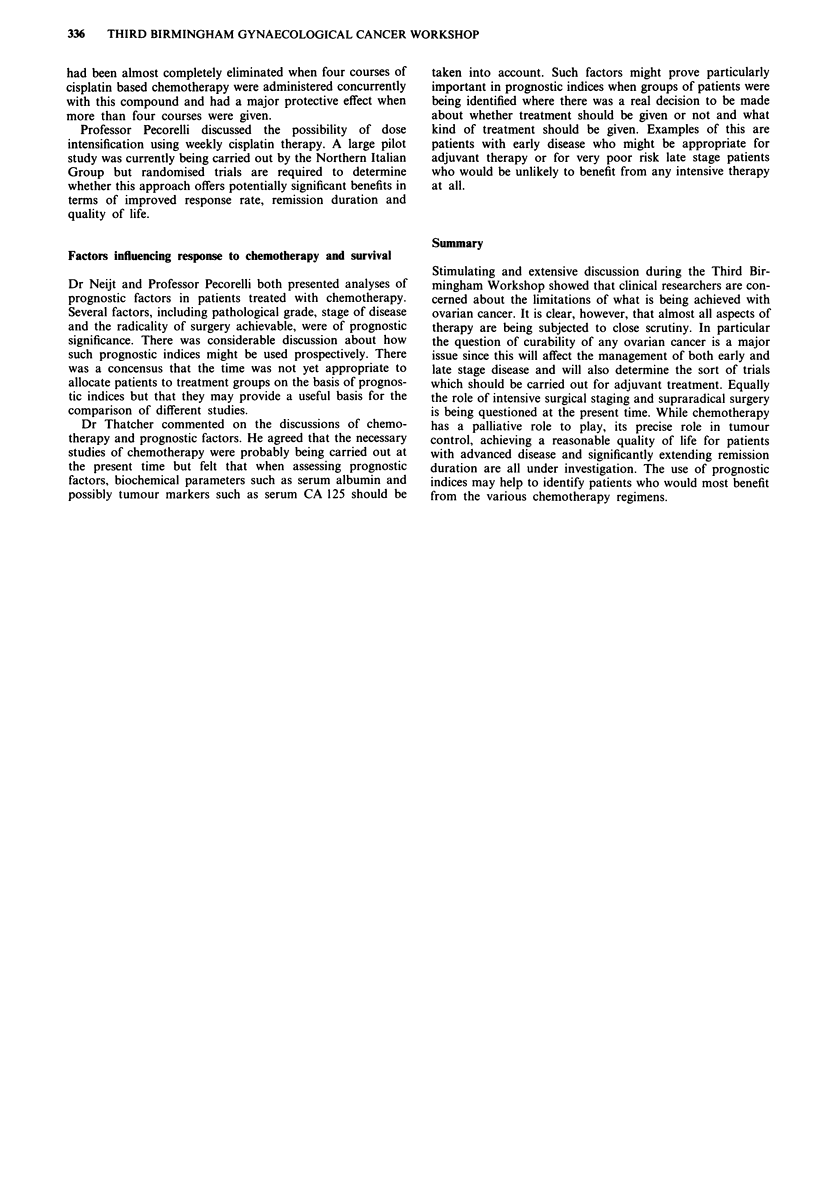# The 3rd Birmingham Gynaecological Cancer Workshop: What are we doing wrong with ovarian cancer?

**Published:** 1990-02

**Authors:** 


Br. J. Cancer (1990), 61, 335-336 © Macmillan Press Ltd., 1990

### The Third Birmingham Gynaecological Cancer Workshop: What are we doing wrong with ovarian cancer?

*Held at Queen Elizabeth Hospital, Edgbaston, Birmingham B15 2TH, UK.*

The outlook for many patients with epithelial ovarian cancer remains poor despite apparent advances in surgery and chemotherapy over the past few decades. The Third Birmingham Gynaecological Cancer Workshop was convened to address specific problems in the management of ovarian cancer and to determine whether new approaches could be adopted.

In an attempt to take a different view of ovarian cancer, speakers from the field presented data which were then challenged by oncological experts from other fields of cancer, Professor Giles from the University of Leeds and Dr Thatcher from the Christie Hospital in Manchester. Over the day discussions were wide ranging but concentrated on several main issues.

### Optimal surgical management

Mr J. Shepherd (St Bartholomew's Hospital and Royal Marsden Hospital, London) addressed the problem of what constituted adequate surgery for ovarian cancer. Complete macroscopic removal of all the tumour if possible was the primary principle of good surgical management but there was disagreement about other procedures that should be carried out. Meticulous staging operations may reveal disease, especially in the retroperitoneum. There was, however, a consensus that extensive retroperitoneal dissection was still of unproven value and was unlikely to make a significant contribution to the welfare of patients.

### Management of stage I and early disease

Mr Shepherd and Professor Pecorelli (Brescia, Italy) presented data on the management of stage I ovarian cancer. In the London series 80% of patients with genuine stage I disease have a 5-year survival without any treatment other than surgical removal. Relapses occur mainly in the first 2-3 years but it was noted by Dr Wiltshaw (Royal Marsden Hospital, London) that relapses were occurring even after 8-10 years. This calls into question the ultimate curability of ovarian cancer and suggests that its behaviour in the long-term may be like that of breast cancer, where the data would suggest that few if any patients can ultimately be said to be cured of their disease.

Professor Pecorelli then presented data on patients who had been managed with conservative surgical treatment for stage I disease. For a variety of reasons, usually the wish to preserve fertility, only the malignant ovarian cyst had been removed. Survival in this group of patients was equivalent to that in populations managed by conventional surgery. In further studies the 5-year survival of 65 patients with stage IA grade I tumours who had received no additional therapy was 100%. Eighty stage IA and IB patients with grade 2/3 tumours were randomised to observation following laparotomy or six cycles of cisplatinum. There was no difference in relapse rate (10% in each arm had been observed so far). One hundred and eight patients with stage IC disease had been randomised to receive either cisplatinum or intraperitoneal radiocolloid. Relapse rates of 30% and 26% had been noted. The ultimate impact on survival for such treatments had not yet been established because of relatively short follow-up. The role of systemic adjuvant treatment therefore is still subject to considerable debate. Salvage therapy may prove to be very effective, especially if relapse or progression is detected at an early stage, and it was agreed that the role of adjuvant systemic therapy required further testing. Trials are in the process of being set up both in the UK and also on a European basis.

The contribution of surgery in advanced disease was discussed. The amount of gross tumour removed appears to influence survival but it was agreed that the ability to debulk optimally may be associated with intrinsic factors of the disease rather than surgical technique alone. The role of surgery in this setting is under re-evaluation at the present time. Second look laparotomy, that is surgery performed at the end of primary chemotherapy, has been demonstrated to have no significant impact on survival in advanced disease. The role of surgery following two or three courses of cytoreductive surgery is now being investigated and only randomised studies will determine the true impact of surgery in this setting.

Professor Giles reviewed surgical management. He acknowledged that surgical staging *per se* was unlikely to have a major influence on survival. He questioned the definition of adequate local control and pointed out in ovarian cancer that a reasonable definition of local disease would be disease within the peritoneum. He urged that all methods of local extirpation should be considered, with approaches such as phototherapy and laser destruction being potential means of controlling disease within the peritoneum. Professor Giles felt that the interaction between systemic treatment and surgical intervention required further investigation and such clinical experiments were urgently needed.

### Chemotherapy

The discussion of chemotherapy was introduced by Dr Wiltshaw. In a review of patients treated at the Royal Marsden Hospital over nearly 20 years she demonstrated that the introduction of cisplatin into treatment protocols had had a major impact on survival and this had been one major factor influencing outcome in patients with advanced disease (FIGO stages III and IV). It remained the case, however, that despite dose intensification and the use of combination therapy, extension of life with effective chemotherapy remained the norm and ultimate cure was unlikely in this group of patients. Further dose intensification using high dose carboplatin did appear to be improving the outlook for patients with stage IV disease in whom the prognosis was particularly poor, and a randomised trial is underway in earlier stage disease to see if cures might result.

### Toxicity of chemotherapy

The toxicity of drugs such as the platinum compounds remained a major problem. Neurotoxicity is one of the major dose limiting effects of cisplatin and can be seen with carboplatin, especially when used in combination with cisplatin. Dr Neijt (Utrecht, Netherlands) reported on the development of a neuropeptide ORG2766 which appears to protect against the problems of neurotoxicity. In a randomised study carried out by the Netherlands Ovarian Cancer Group neurotoxicity

Sponsored by the Cancer Research Campaign. Supported with additional grants from Boehringer Ingelheim, Sterling Drug and Farmitalia.
Report written by R. Varma and G. Blackledge. Workshop organised by G. Blackledge, C. Redman and J. Buxton.

had been almost completely eliminated when four courses of cisplatin based chemotherapy were administered concurrently with this compound and had a major protective effect when more than four courses were given.

Professor Pecorelli discussed the possibility of dose intensification using weekly cisplatin therapy. A large pilot study was currently being carried out by the Northern Italian Group but randomised trials are required to determine whether this approach offers potentially significant benefits in terms of improved response rate, remission duration and quality of life.

**Factors influencing response to chemotherapy and survival**

Dr Neijt and Professor Pecorelli both presented analyses of prognostic factors in patients treated with chemotherapy. Several factors, including pathological grade, stage of disease and the radicality of surgery achievable, were of prognostic significance. There was considerable discussion about how such prognostic indices might be used prospectively. There was a concensus that the time was not yet appropriate to allocate patients to treatment groups on the basis of prognostic indices but that they may provide a useful basis for the comparison of different studies.

Dr Thatcher commented on the discussions of chemotherapy and prognostic factors. He agreed that the necessary studies of chemotherapy were probably being carried out at the present time but felt that when assessing prognostic factors, biochemical parameters such as serum albumin and possibly tumour markers such as serum CA 125 should be taken into account. Such factors might prove particularly important in prognostic indices when groups of patients were being identified where there was a real decision to be made about whether treatment should be given or not and what kind of treatment should be given. Examples of this are patients with early disease who might be appropriate for adjuvant therapy or for very poor risk late stage patients who would be unlikely to benefit from any intensive therapy at all.

**Summary**

Stimulating and extensive discussion during the Third Birmingham Workshop showed that clinical researchers are concerned about the limitations of what is being achieved with ovarian cancer. It is clear, however, that almost all aspects of therapy are being subjected to close scrutiny. In particular the question of curability of any ovarian cancer is a major issue since this will affect the management of both early and late stage disease and will also determine the sort of trials which should be carried out for adjuvant treatment. Equally the role of intensive surgical staging and supraradical surgery is being questioned at the present time. While chemotherapy has a palliative role to play, its precise role in tumour control, achieving a reasonable quality of life for patients with advanced disease and significantly extending remission duration are all under investigation. The use of prognostic indices may help to identify patients who would most benefit from the various chemotherapy regimens.